# A Novel Approach for Screening Sericin-Derived Therapeutic Peptides Using Transcriptomics and Immunoprecipitation

**DOI:** 10.3390/ijms24119425

**Published:** 2023-05-29

**Authors:** Riyuan Wang, Yuancheng Wang, Jianxin Song, Huanhuan Tan, Chi Tian, Dongchao Zhao, Sheng Xu, Ping Zhao, Qingyou Xia

**Affiliations:** 1 Integrative Science Center of Germplasm Creation in Western China (CHONGQING) Science City & Southwest University, Biological Science Research Center, Southwest University, Chongqing 400715, China; 2 Chongqing Key Laboratory of Sericultural Science, Southwest University, Chongqing 400715, China; 3 Chongqing Engineering and Technology Research Center for Novel Silk Materials, Southwest University, Chongqing 400715, China; 4 Guangxi Engineering Center in Biomedical Materials for Tissue and Organ Regeneration, Guangxi Medical University, Nanning 530021, China

**Keywords:** therapeutic peptide, screening method, sericin, extracellular matrix, collagen, elastin, immunoprecipitation, natural resources

## Abstract

With the demand for more efficient and safer therapeutic drugs, targeted therapeutic peptides are well received due to their advantages of high targeting (specificity), low immunogenicity, and minimal side effects. However, the conventional methods of screening targeted therapeutic peptides in natural proteins are tedious, time-consuming, less efficient, and require too many validation experiments, which seriously restricts the innovation and clinical development of peptide drugs. In this study, we established a novel method of screening targeted therapeutic peptides in natural proteins. We also provide details for library construction, transcription assays, receptor selection, therapeutic peptide screening, and biological activity analysis of our proposed method. This method allows us to screen the therapeutic peptides TS263 and TS1000, which have the ability to specifically promote the synthesis of the extracellular matrix. We believe that this method provides a reference for screening other drugs in natural resources, including proteins, peptides, fats, nucleic acids, and small molecules.

## 1. Introduction

Targeted therapeutic peptides (TTPs) are a series of functional peptides that contain 2–50 amino acids. TTPs have a molecular weight of approximately 0.5–5 kDa [1]. Apart from artificial synthesis, these peptides also implicitly exist in the proteins of animals, plants, bacteria, and fungi, and can be released during enzymatic digestion, food digestion, or microbial action [2]. TTPs have been proven to exhibit some biological activities, including antibacterial, anti-inflammatory, growth-promoting, pro-differentiation, anti-hypertensive, and anti-cancer activities [3,4]. Compared to traditional small-molecule drugs (SMD), TTPs have a larger binding interface with their targets exhibiting higher specificity and robustness, thus avoiding side effects resulting from off-target effects. In addition, TTPs exhibit low immunogenicity due to their uncomplicated spatial conformation. Therefore, TTPs have been used in many medical fields, and more than 100 TTPs have been approved by the FDA to treat diseases such as tissue trauma, heart failure, hypertension, diabetes, and cancer [5,6].

Three conventional methods have been frequently used for screening TTPs in natural proteins: the bioactivity-guided method, the phage display method, and in silico screening method. For the bioactivity-guided screening method, the first step is to divide the enzymatically hydrolyzed crude peptides into products with different molecular weights using membrane filtration technology. The biological activity of each hydrolytic product is analyzed to select the most active component. TTPs in the most active component are isolated and enriched according to the different properties of the peptides, such as molecular weight, polarity, and charge, which are then examined using bioactivity assays. Nong et al. identified a dipeptidyl peptidase-IV inhibitory peptide from the egg yolk hydrolysate of soft-shell turtles [7]. In the phage display method, a DNA library is separately integrated into the genome of different phages to harbor peptides in their tails allowing positive phages to bind to a fixed target. After extended breeding by infecting bacteria, the positive phages are collected to extract the genome for sequencing required for evaluating TTPs. The EBD fibronectin peptide for targeting prostate cancer was discovered in this way [8]. In the in silico screening method, the amino acid sequence of the proteins was virtually digested in silico to generate a peptide library. Peptide candidates with highly similar spatial structures and physicochemical properties to those in the database were theoretically simulated and experimentally synthesized to determine their biological activity. Tejano et al. used the BIOPEP-UWM database to screen peptides in the Chlorella sorokiniana protein [9]. All conventional techniques mentioned above have played an important role in the discovery of TTPs. However, these methods have drawbacks in performing numerous experiments and complex screening processes, in addition to long screening times, which in turn restricts the innovation and clinical translation of peptide drugs due to inefficient screening processes.

Except for the antimicrobial peptides that directly act on the cell membrane of microbes, most TTPs bind to receptors or ion channels through intermolecular forces, such as van der Waals forces, hydrogen bonds, and salt bridges. Extracellular signals originate from the TTP-target complex, enabling cells to respond to external stimuli. For example, glucagon-like peptide 1 (GLP1), a target therapeutic peptide in the treatment of diabetes, binds to the GLP1 receptor on the surface of islet cells and then transmits signals to the nucleus via the insulin signaling pathway to activate insulin secretion and inhibit glucagon release [10]. This process balances metabolism in the body but consumes the receptors and signal transduction molecules of the islet cells [11,12,13]. To maintain proper cellular function, cells transcribe genes associated with these molecules and translate them to replenish the missing signaling protein. Therefore, when the transcription of cells treated with a peptide library of natural proteins is clear, the TTP-triggered signaling pathways and their receptors are easily determined. A cell receptor is the target of a ligand and the interaction between them is specific and stable. Once the active center of a receptor binds to a suitable ligand, it forms a stable complex [14]. Hence, as soon as this receptor-ligand complex is captured, the TTPs can be screened.

Co-immunoprecipitation (CoIP) is an effective tool for studying protein–protein interactions based on the specificity between antibodies and antigens [15]. CoIP comprises the following three steps as follows: (1) the antibody is cross-linked with the magnetic beads to form a complex that can be fixed with magnets; (2) those fixed complexes can capture another complex formed by antigens and unknown proteins to generate a final complex; (3) the final complex is washed and detected by SDS-PAGE, Western blot (WB), or other analytical techniques. The results obtained by CoIP have high accuracy because the interacting proteins are natural proteins that naturally combine with each other by non-covalent bonding. If a different detection method such as liquid chromatography–mass spectrometry (LC-MS), gas chromatography –mass spectrometry (GC-MS), or other modern sequencing techniques is used for CoIP, studying the interaction between receptors and ligands, including proteins, lipids, nucleic acids, and small-molecule drugs, becomes more feasible.

Sericin, one of the major components of the silkworm cocoon, is a group of natural macromolecular glycoproteins with a molecular weight of approximately 20–400 kDa. It is synthesized and secreted by the middle silk glands of silkworms and covers the surface of the silk fibroin [16]. Previous studies have demonstrated that sericin and its hydrolysate have many biological activities, such as cell proliferation and synthesis of extracellular matrix proteins [17,18], which are beneficial for curing illnesses such as wound repair and colitis treatment [19,20]. In this study, a novel strategy for screening TTPs in the raw material of sericin was developed based on transcriptomics and co-immunoprecipitation techniques. The proposed strategy is based on the construction of peptide libraries, screening of TTP-triggered signaling pathways, CoiP, and identification and functional analysis of target peptides. The results showed that the two most active and selective peptides were successfully screened and could significantly promote the transcription and synthesis of extracellular matrix proteins. Our proposed strategy can also be utilized for screening other drug candidates, including proteins, lipids, nucleic acids, and small-molecule drugs.

## 2. Results

### 2.1. Construction of Sericin Peptide Library and Its Promotion of Extracellular Matrix Synthesis in HaCaT Cells

**Figure 1:**
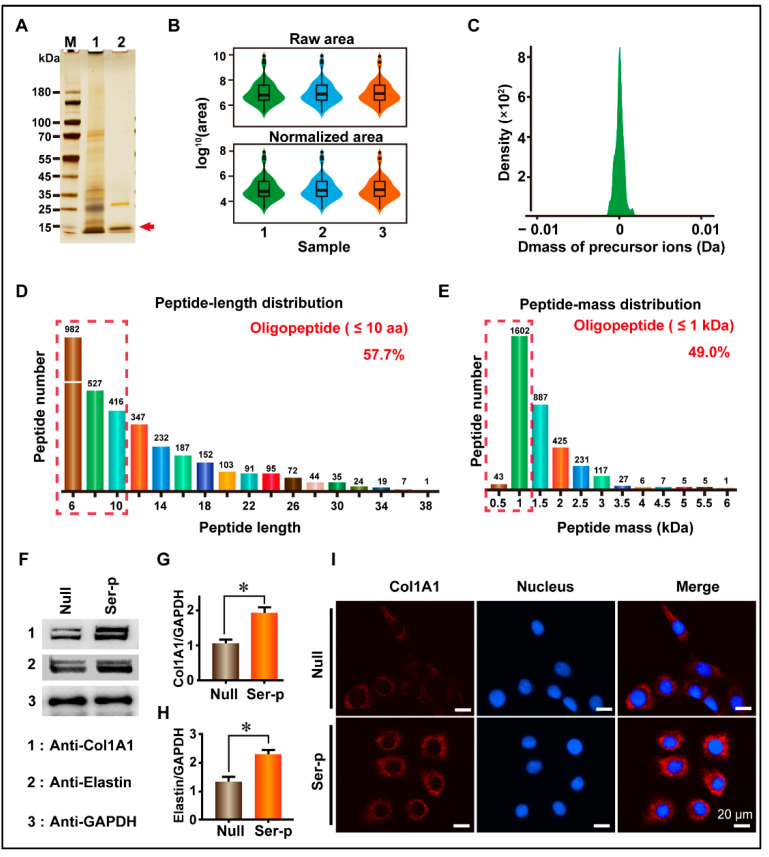
Establishment of sericin peptide library and detection of its biological activity. (**A**) SDS-PAGE detection of a sericin peptide library. 1 and 2 represent sericin extracted using Tris-HCl and sericin peptide library after enzymes treatment, respectively. (**B**) Distribution map of protein abundance values between repeated samples. (**C**) Tolerance distribution of parent-ions mass in the sericin peptide library. (**D**) Amino acid number distribution of peptides. Oligopeptides with an amino acid number less than 10 are marked with a red dotted box. (**E**) The molecular weight distribution of peptides. Oligopeptides with molecular weight less than 1 kDa are marked with a red dotted box. (**F**) The Sericin peptide library promotes the synthesis of extracellular matrix proteins. The molecular weights of Col1A1 and elastin are ~200 kDa and ~68 kDa, respectively. (**G**,**H**) Comparison of Col1A1 and elastin protein content in culture medium. (**I**) Comparison of immunofluorescence signal. (Scale bar, 20 μm). All experiments were conducted with three independent replicates, and data were expressed as the mean ± standard error of the mean (SEM, error bar). For the significance test: * *p* < 0.05, versus Null group.

To establish and characterize the sericin peptide library, sericin treated with proteases was collected and tested using SDS-PAGE and LC-MS. The SDS-PAGE results showed that the high molecular weight sericin was degraded into peptides with molecular weights less than 15 kDa (Figure 1A). The LC-MS results showed that the shape of the box diagram, which represents the protein abundance values, was maintained among three repeated experimental samples, and there was no significant difference after normalization (Figure 1B). The tolerance distribution of these peptides between the actual molecular weight and theoretical molecular weight was close to 0. These results illustrate that the sericin peptide library was successfully constructed and could be easily replicated. Peptides in this library were distributed in the 6–38 and 0.5–6 kDa region, in which oligopeptides with amino acid number ≤ 10 or molecular weight ≤ 1 kDa were the main components accounting for 59.9% and 49.0%, respectively (Figure 1D,E).

To validate the ability of the sericin peptide library to promote the synthesis of extracellular matrix proteins, the sericin peptide library was added to a fresh DMEM medium supplemented with 10% FBS to culture HaCaT cells. Collagen I and elastin are two important components of extracellular matrix proteins that are synthesized and secreted into the extracellular matrix. Thus, the culture medium was collected to detect collagen I and elastin, and the cells were fixed for immunofluorescence observation. WB results showed that the gray value of collagen I and elastin in the Ser-P group medium was 1.91 ± 0.11 and 2.30 ± 0.10, respectively, which were 72.07% and 72.93% higher than those in the Null group (1.11 ± 0.09 and 1.33 ± 0.13 for collagen I and elastin, respectively) (Figure 1F–H, Table 1). Similarly to the WB results, immunofluorescence results showed that the extracellular collagen signal of the Ser-P group was significantly stronger than that of the Null group (Figure 1I). These results indicate that the sericin peptide library can promote the synthesis of extracellular matrix proteins by HaCaT cells.

(1) $$\mathrm{Ratio}\;=\;\frac{\mathrm{Gray}\;\mathrm{value}(\mathrm{extracellular}\;\mathrm{matrix}\;\mathrm{protein})}{\mathrm{Gray}\;\mathrm{value}(\mathrm{GAPDH})}$$

### 2.2. Sericin Peptide Library Promotes Transcription of the Extracellular Matrix by Regulating the TGF-β Signaling Pathway

**Figure 2:**
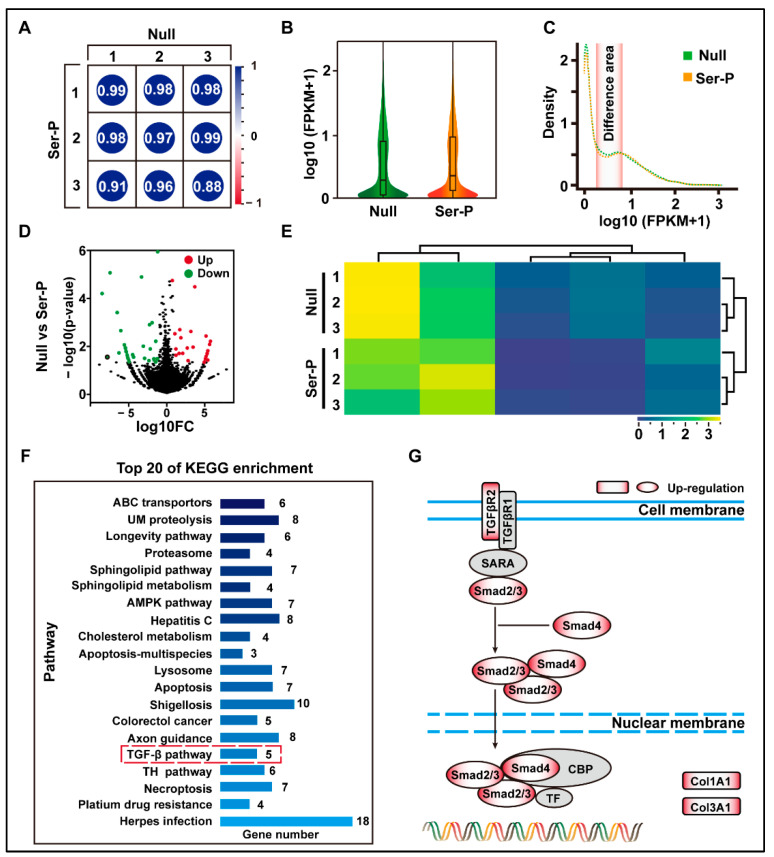
Transcriptome analysis of HaCaT cells after sericin peptide library treatment. (**A**) The gene expression level FPKM was selected to evaluate the Pearson correlation analysis between samples. (**B**) Analysis of gene expression of the normalized sample. (**C**) The density distribution curve of gene expression. The differentiated areas are marked in pink. (**D**) Volcano map of gene expression differences between Null and Ser-P group. Dots with red, green, and black represent upregulated, downregulated, and not significantly changed genes assessed by hard thresholds, which have two standards: |log2FC| > 1 and −log10 *p* value > 1.3. (**E**) Hierarchical clustering heat map of differential genes between Null and Ser-P group. The columns and rows represent the different samples and different expressed genes, respectively. (**F**) Top 20 of KEGG pathway enrichment analysis. TGF-β signaling pathway related to extracellular matrix synthesis is marked with a red dotted box. UM proteolysis refers to ubiquitin-mediated proteolysis. (**G**) Gene upregulating expression in classical TGF-β pathway in Ser-p group. All experiments were conducted with three independent replicates and data were expressed as log10 (FPKM).

To understand the effect of the sericin peptide library on cell transcription, cells in the Null and Ser-p groups were collected for transcriptomic analysis at 24 h. The transcriptomic results showed that the Ser-P group had significant differences in total gene transcription, density distribution, and change amplitude compared to the Null group (Figure 2A–E). After enrichment and analysis of these differential genes by KEGG, twenty signaling pathways were upregulated in the Ser-p group compared to the Null group, including ABC transporters, ubiquitin-mediated proteolysis, and longevity pathway. Among these upregulated signaling pathways, only the TGF-β signaling pathway was involved in the synthesis of extracellular matrix proteins (Figure 2F). Further analysis revealed that genes encoding TGF-β type 2 receptor (TGFβR2), Smad2, Smad4, Col1A1, and Col3A1 were upregulated in the classical TGF-β signaling pathway (Figure 2G). These results suggested that some TTPs in the sericin peptide library may regulate the TGF-β signaling pathway by binding to the TGF-β type II receptor, hence promoting the synthesis of extracellular matrix proteins.

### 2.3. Targeted Screening of TTPs Based on CoIP Technology

**Figure 3:**
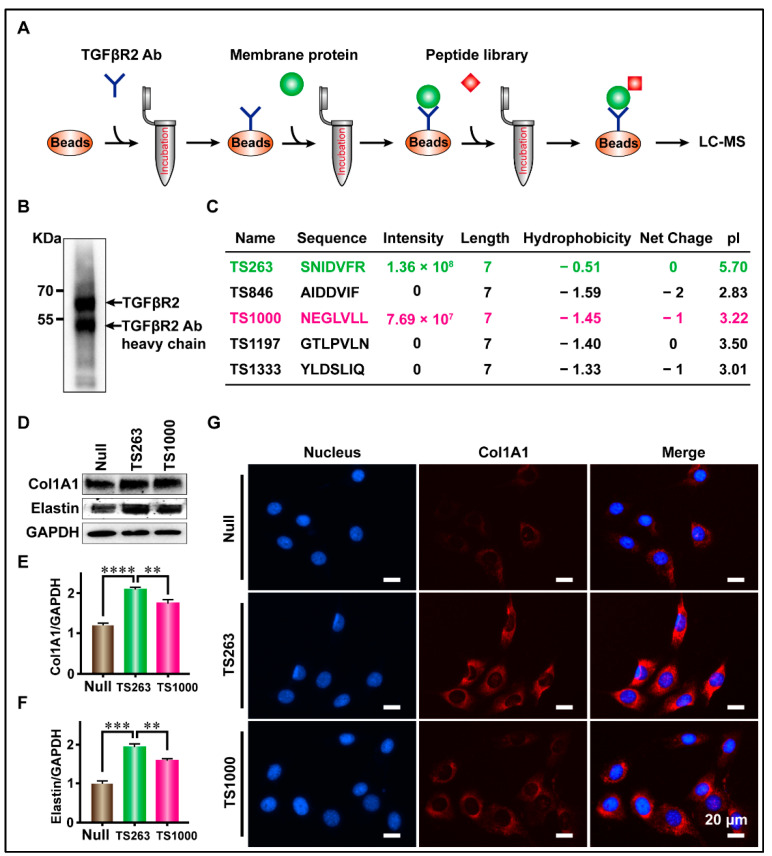
Targeted screening for TTPs that promote extracellular matrix synthesis. (**A**) Schematic diagram of the screening process. (**B**) The capture of TGFβR2 receptor. (**C**) Peptides captured by CoIP. (**D**) Detection of the major components of extracellular matrix in culture medium. (**E**,**F**) Comparison of Col1A1 and Elastin protein content in culture medium. (**G**) Detection of cellular immunofluorescence signal after TPP treatment. The data was presented as the mean ± SEM. For significant: ** *p* < 0.01, *** *p* < 0.001, **** *p* < 0.001, versus TS263 group.

Lau et al. reported that more than 90% of peptides undergoing clinical development are receptor targeted [21]. Therefore, we used CoIP technology to screen TTPs targeting TGFβR2, and the screening results were analyzed using SDS-PAGE, WB, and LC-MS. WB results showed that the TGFβR2 antibody, fixed by protein G magnetic beads, successfully captured TGFβR2 in total CMP (Figure 3B). LC-MS results showed that a total of five TTPs were identified, where only TS263 and TS1000 had iBAQ intensity of 1.36 × 10^8^ and 7.69 × 10^7^, while that of other TTPs was measured at 0 (Figure 3C). The basic characteristics of these two TPPs are as follows: the number of amino acids (7); hydrophobicity (−0.51 and −1.59); net charge at pH = 7.0 (0 and −1); isoelectric point (5.7 and 3.22) (Figure 3C). WB and immunofluorescence results showed that TS263 and TS1000 could promote the synthesis of extracellular matrix proteins, and the gray value ratio of collagen I and elastin were calculated at 2.08 ± 0.03 and 1.94 ± 0.05 in the TS263 group, which was 19.78% and 22.18% higher than 1.73 ± 0.05 and 1.59 ± 0.03 in the TS1000 group (Figure 3D–F; Table 2).

**Figure 4:**
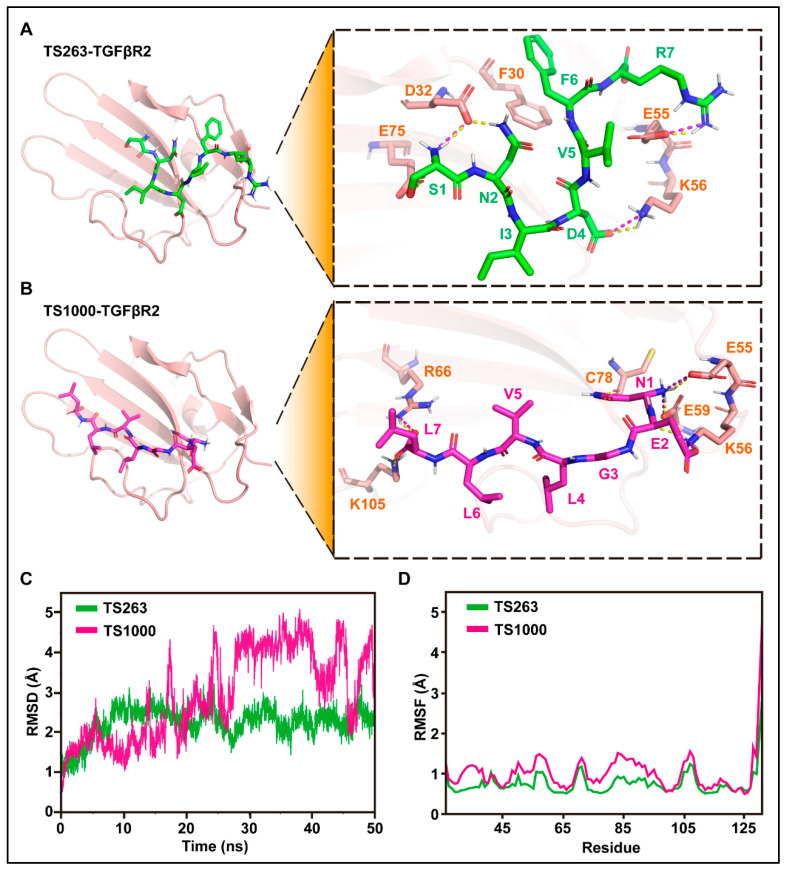
Molecular docking and its dynamics simulation. (**A**,**B**) Prediction of amino acid interactions between TS263, TS1000, and TGFβR2. H-bonds and salt bridges were separately represented by yellow dashed lines and wine-red dashed lines. (**C**) Stability analysis of the interaction between TS263, TS1000, and TGFβR2. (**D**) Flexibility analysis of the interaction between TS263, TS1000, and TGFβR2.

The interaction site, molecular dynamics, and binding free energy between TS263/1000 ligand and TGFβR2 receptor were predicted using molecular docking technology. The docking results showed that H-bonds exist between S1, N2, D4, and R7 in TS263 and D32, K56, and E55 in TGFβR2, and the salt bridge appears between S1, D4, and R7 in TS263 and D32, K56, and E55 in TGFβR2 (Figure 4A), while TS1000 has three amino acids (N1, E2, and L7) that interact with the receptor via H-bond or salt bridge (Figure 4B). Unlike TS1000, which interacts with the TGFβR2 receptor only in the N-terminus and C-terminus of its spatial conformation, TS263 interacts with the TGFβR2 receptor in the N-terminus, central region, and C-terminus of its spatial conformation, resulting in a stronger interaction. In addition, the value of the root mean square deviation (RMSD) and the stability of root mean square fluctuation (RMSF) of TS263 calculated from the receptor-binding process was smaller and more stable than that of TS1000 (Figure 4C,D). The binding free energy, which is negatively related to the affinity between the ligand and receptor, was computed at −22.26 ± 2.22 kcal/mol in the TS263_TGFβR2 complex, which was lower than the values calculated for the TS1000_ TGFβR2 complex (−15.52 ± 2.55 kcal/mol) (Table 3). These results demonstrate that both TS264 and TS1000 can bind to the TGFβR2 receptor; however, the interaction between TS263 and the TGFβR2 receptor is stronger than that between TS1000 and TGFβR2.

### 2.4. TTP Regulates the TGF-β Signaling Pathway to Promote Extracellular Matrix Synthesis in HaCaT Cells

**Figure 5:**
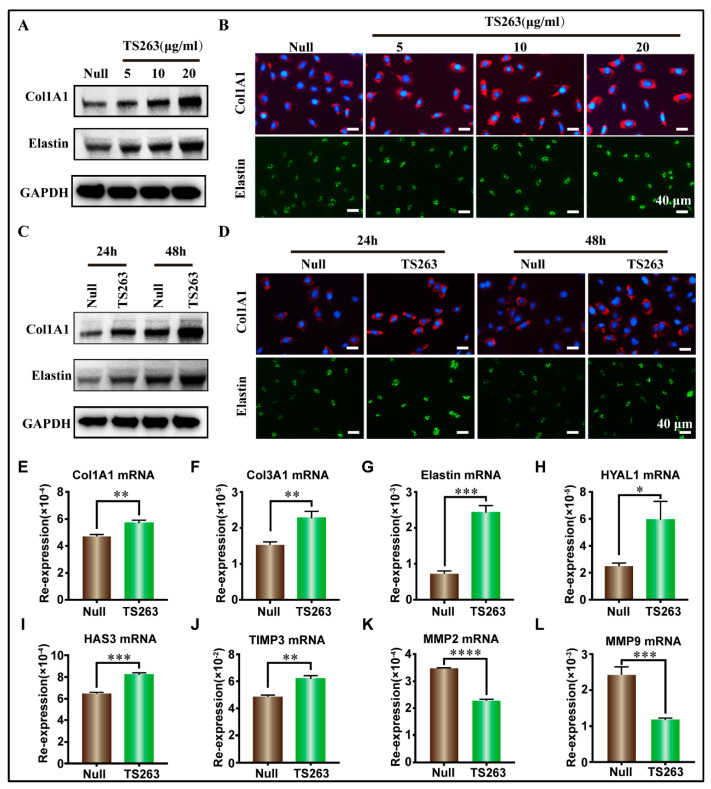
TS263 promotes the synthesis of extracellular matrix protein. (**A**,**B**) TS263 promotes HaCaT cells to synthesize extracellular matrix protein in a concentration-dependent manner. (Scale bar 40 μm). (**C**,**D**) TS263 promotes HaCaT cells to synthesize extracellular matrix protein in a time-dependent manner. (Scale bar 40 μm). (**E**–**L**) RT-PCR analysis of genes promoting extracellular matrix synthesis or degradation. Experiments were independently performed in triplicates, and data are presented as the mean ± SEM (error bar). For the significance test: * *p* < 0.05, ** *p* < 0.01, *** *p* < 0.001, **** *p* < 0.001 versus Null group.

TS263 was selected as a target to verify its biological activity because it can promote the synthesis of extracellular matrix proteins. WB results revealed that TS263 promoted the synthesis of extracellular matrix proteins in HaCaT cells and HFF-1 cells in a concentration- and time-dependent manner (Figure 5A–D, Figure S1A–C). RT-PCR results showed that TS263 enhanced the transcription of Col1A1, Col3A1, elastin, collagen synthase 1 (HYAL1), hyaluronan synthase 3 (HAS3), and metalloproteinase inhibitor protein 3 (TIMP3) genes, and inhibited the transcription of metalloproteinase 2 (MMP2) and metalloproteinase 9 (MMP9), which are capable of degrading extracellular matrix proteins (Figure 5E,L; Table 4).

**Figure 6:**
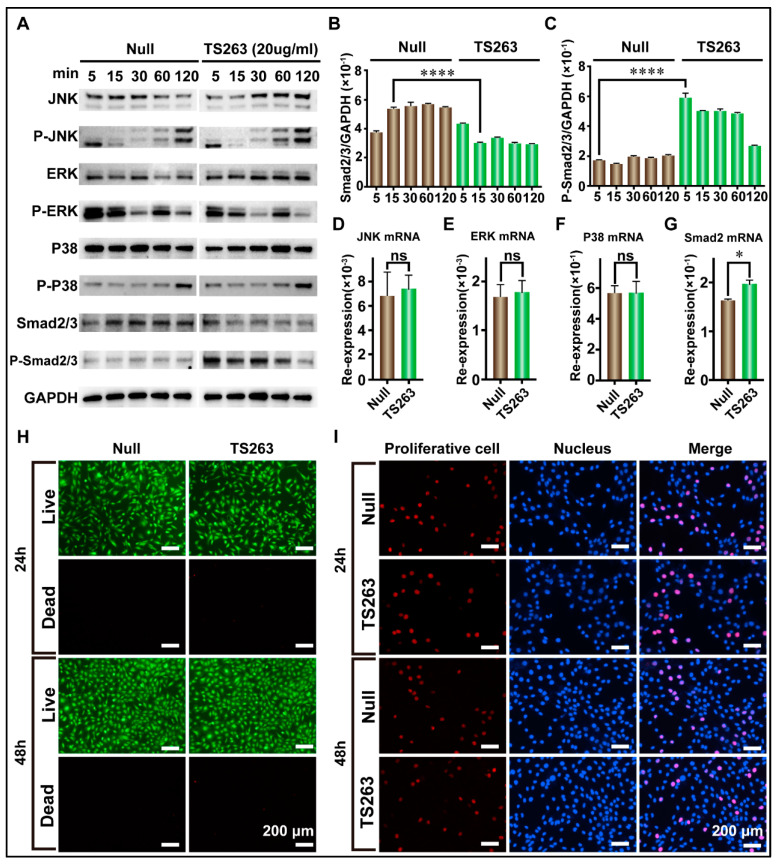
TS263 precisely regulates the TGF-β signaling pathway. (**A**) WB analysis of the key factor in JNK, ERK, P38, and TGF-β signaling pathway. The molecular weights of JNK, ERK, P38 and smad2/3 are ~46 kDa and~54 kDa (two bands), ~42 kDa and ~44 kDa (two bands), ~38 kDa, and ~60 kDa, respectively. (**B**,**C**) Comparison of the protein abundance of Smad2/3 and P-Smad2/3. (**D**–**G**) RT-PCR analysis of the key gene in JNK, ERK, P38, and TGF-β signaling pathway. Re-expression refers to relative expression. (**H**) Live and dead analysis of cell viability. (**I**) EdU staining for detecting cell proliferation. The data was presented as the mean ± SEM (error bar). For significant: * *p* < 0.05, **** *p* < 0.001, versus Null group. ns, not significant.

To the best of our knowledge, cell proliferation can also promote the synthesis of extracellular matrix proteins. Therefore, the para signal pathway, which cooperates with the TGF-β signaling pathway to promote cell proliferation, was selected for further investigation. The RT-PCR results showed that TS263 only promoted the transcription of the Smad2 gene but had no effect on the JNK, ERK, and P38 genes (Figure 6D,G; Table 5). The WB results showed that the total protein or its phosphorylated forms of JNK, ERK, and P38 in the TS263 group did not change dramatically compared to the Null group (Figure 6D,G), while the total protein of Smad2/3 decreased and its phosphorylated form increased (Figure 6A,C). Live and dead cells and EdU staining showed no significant difference in cell numbers and proliferation capacity between the Null and TS263 groups. Therefore, we concluded that TS263 acts only on the classical TGF-β signaling pathway and does not trigger other signaling pathways.

In conclusion, TS263 upregulated the TGF-β signaling pathway to promote the synthesis of extracellular matrix proteins.

## 3. Discussion

TTPs are used in the treatment of many diseases worldwide, including hypertension, diabetes, and oncology, because of their specificity, high safety, and low price. The preliminary pharmaceutical market analysis stated that the sales of TTP drugs exceeded $50 billion in 2019, accounting for approximately 5% of the global pharmaceutical market and that percentage increases annually [22]. Therefore, the development of TTP drugs is a hot research topic that has attracted the attention of many researchers. 

Sericin, as a natural product, is often processed as a scaffold, drug carrier, biological tracer, or directly used as a wound dressing because of its good moisturizing property, low immunogenicity, good biocompatibility, and biodegradability [23]. However, the high-molecular-weight sericin proteins obtained using the traditional methods maintain their advanced structure, and the content of TTPs is not effectively released, resulting in fewer specific pharmaceutical properties [24]. Therefore, we created trypsinogen-based self-degummed silk in the early stage, which allows us to easily obtain sericin peptides [17]. However, we found that the self-degummed sericin can be completely separated from the silk fibroin, but it cannot be fully degraded into low-molecular-weight peptides, which would affect the filtration of the sericin solution. Hence, in this study, we first collected the self-degummed sericin peptides with lower molecular weights and retained their cell proliferation-promoting properties. Subsequently, the remaining sericin peptides with large molecular weights were further digested by chymotrypsin. The construction of a sericin peptide library using this stepwise enzymatic method can not only reduce the loss of sericin protein and greatly improve its utilization rate but also make the sericin peptide library exhibit the characteristics of low molecular weight peptides. In the enzymatically generated sericin peptide library, the content of oligopeptides with amino acids <10 is approximately 57.7%, and the content of oligopeptides with molecular weight <1 kDa is approximately 49.0%, which is within the range of skin absorption. In addition, it can promote cell proliferation and extracellular matrix protein synthesis. This enzymatic sericin peptide library can be potentially used to produce anti-aging agents or adjuvants for the skin [25,26].

The use of traditional methods to screen TTPs from enzymatic peptide libraries in natural resources is a complex, time-consuming, and inefficient process, which hinders the development of new drugs. Comparing the three conventional screening approaches, we found that previous researchers have made little or no use of the working principle of TTP: ligand > receptor > intracellular signal > cellular transcription and translation > protein [27]. Under normal circumstances, cells can compensate for missing proteins through gene transcription and translation. Therefore, if we comprehensively investigate the changes in cell signaling pathways, we can easily determine the receptor and signaling pathways activated by TTPs. Transcriptomics or translatomics is an effective method to accurately reflect changes in intracellular mRNA or protein levels [28,29]. However, the former is more commonly utilized by researchers because of its well-developed technology, short test cycle, and low price. Therefore, we innovatively combined transcriptomics with CoIP technology to establish a simple and effective screening method that allows us to easily screen TS263/1000 from an enzymatic sericin peptide library that can promote the synthesis of extracellular matrix proteins. This can effectively avoid (1) the problems of performing too many validation experiments in the bioactivity-guided method, (2) the complexity of constructing the library in the phage display method, and (3) excessive false positives in the virtual screening method in silico. In addition, the receptors used in this method are directly derived from cells, which saves time and the cost required for recombinant expression, maintains their natural activity, and greatly improves the efficiency of screening pharmaceutical peptides.

The specificity of peptide drugs is also one of the important factors in determining their use for disease treatment. Off-targeting drugs can cause some unpredicted side effects [30]. The TGF-β signaling pathway can regulate many cellular processes, including growth, differentiation, and extracellular matrix synthesis [31]. The TGF-β signaling pathway can be divided into two types: classical and non-classical pathways [32]. The non-classical TGF-β signaling pathways always cooperate with three para-signaling pathways to promote cell proliferation, including the JNK, ERK, and P38 signaling pathways [33]. Therefore, in our study, we validated the specificity of TS263. The key factors of the four signaling pathways mentioned above were considered to investigate the levels of transcription, translation, and phosphorylation of Smad2/3, JNK, ERK1/2, and P38. TS263 has been suggested to only promote the transcription and phosphorylation of Smad2/3 through the TGFβR2 receptor, but it cannot affect the transcription and phosphorylation of JNK, ERK, and P38. We also examined cell viability and proliferation in the Null and TS263 groups and found no significant differences between them. Therefore, we believe that TTPs screening using our approach can specifically regulate cellular processes.

## 4. Methods and Materials

### 4.1. Cell Culture

Human immortal keratinocytes (HaCaT) and human foreskin fibroblasts (HFF-1) were purchased from Procell Life Science & Technology Co., Ltd. (Wuhan, China) and cultured in a DMEM medium supplemented with 10% fetal bovine serum (Gibco, New York, NY, USA) at 37 °C in an incubator with 5% CO_2_.

### 4.2. Preparation of Sericin Peptide Library

Ten grams of self-degummed silk were immersed in 500 mL of 1 mM of Tris-HCl solution for 24 h. The supernatant was then collected by centrifugation at 1.8 × 10^4^ rpm and heated at 121 °C for 30 min to inactivate trypsin activity. Finally, the sericin solution was desalted using a dialysis bag with a molecular retention of 500 kDa and filtered through a 0.22 µm membrane. The remaining sericin on the membrane with a higher molecular weight was collected and reprocessed with chymotrypsin heated, desalted, and filtered. The collected solution was mixed, freeze-dried, and stored at −80 °C.

### 4.3. Sericin Peptide Library Promotes Transcription and Synthesis of Extracellular Matrix Proteins 

When cell fusion reached 70–80% in the tissue culture dish, HaCaT cells or HFF-1 cells were re-suspended with trypsin (Gibco, New York, NY, USA) and planted in a 24-well plate at a density of 1.0 × 10^4^ cells/well. These cell-containing wells were divided into Null and Ser-p groups and replaced with 200 μL of fresh DMEM medium with 10% FBS after 12 h. After treating each well of the Ser-p group with a 0.4 μg sericin peptide library for 48 h, the medium was collected, and the cells were lysed with RIPA (Beyontime, Shanghai, China).

### 4.4. SDS-PAGE and Western Blotting 

Medium or cell lysates containing 20 ug of protein were separated on a polyacrylamide gel with a gradient of 4–20% (Gencript, Nanjing, China) and then transferred to PVDF membranes. After blocking with 5% skim milk solution, washing with 1× TBST, incubation with a primary antibody, washing with 1× TBST, incubation with a secondary antibody, and washing with 1× TBST, the membrane was transferred to a chemiluminescence imaging system (Champchemi 610 plus, Clinx, Shanghai, China) to record the signal.

The primary antibodies used in this study were anti-Col1A1, anti-Elastin, anti-TGFβR2, anti-Smad2/3, anti-phosphorylated-Smad2/3, anti-ERK1/2, anti-phosphorylated-ERK1/2, anti-JNK, anti-phosphorylated-JNK, anti-P38, anti-phosphorylated-P38, and anti-GAPDH. All antibodies were purchased from CST except for anti-elastin (Abcam, Cambridge, UK). The secondary antibody used was goat anti-rabbit IGG (Beyotime, Shanghai, China). The primary and secondary antibodies were diluted with 1× TBST at a ratio of 1:10,000 (*v*/*v*).

### 4.5. Transcriptome Analysis and Reverse Transcription Polymerase Chain Reaction (RT-PCR)

Total rRNA was extracted as previously described [34]. Cells treated with sericin or TTP were collected and washed with 1× PBS. Total RNA was extracted using Total RNA Kit I (Omega, New Orleans, LA, USA) according to the manufacturer’s instructions.

For transcriptome analysis, 1 μg of mRNA was enriched using oligo (dT) to construct the sequencing library, followed by sequencing. Data were recorded and analyzed by Qiantang Biotechnology Co., Ltd. (Suzhou, China). 

For reverse transcription polymerase chain reaction (RT-PCR), 1 μg of mRNA was inverted to cDNA using a TransScript^®^ II All-in-One First-Strand cDNA Synthesis SuperMix Kit (Transgen, Beijing, China), and RT-PCR was performed using an ABI Prism 7500 sequence detection system (ABI, Waltham, MA, USA). Primers used in this study are listed in Table 6.

### 4.6. Immunofluorescence Staining

After removal of the culture medium, cells were processed using the following steps: washed with 1× PBS, fixed with 4% paraformaldehyde for 15 min, neutralized with 1× TBST solution containing glycine at a concentration of 1 mg/mL, blocked with 1×TBST solution containing 5% goat serum for 2 h, incubated with a primary antibody for 2 h, washed with 1× TBST, and incubated with a secondary antibody for 2 h. After washing twice with 1× TBST, the cells were placed on an EVOS FL AUTO fluorescence microscope (Thermo Fisher Scientific, Waltham, MA, USA) to record the fluorescence signal.

The primary antibodies used in this experiment were anti-Col1A1 and anti-elastin, whereas the secondary antibody was a goat anti-rabbit IGG. The dilution ratio of the antibodies was 1:500.

### 4.7. Co-Immunoprecipitation (CoIP)

Cell membrane proteins (CMP) were extracted from HaCaT cells using the Minute™ Plasma Membrane Protein Isolation and Cell Fractionation Kit (Invent, Eden Prairie, MN, USA) and stored at −80 °C. Subsequently, 5 ug of anti-TGFβR2 antibody and protein G beads (Dynabeads, Thermo Fisher, Waltham, MA, USA) were co-incubated for 2 h and then cross-linked using bis (sulfosuccinimidyl) suberate for 15 min. After termination with 1 M Tris-HCl, the complex was washed with 1 × TBST and incubated with CMP overnight. The supernatant was removed, and the sample was incubated with 500 μL of sericin at a concentration of 1 mg/mL for 2 h. After washing with 1× PBS, the sample was dissolved in 5× SDS-PAGE loading buffer, with or without R250 dye, and heated in a water bath at 100 °C for 10 min. Finally, the supernatant was collected to perform SDS-PAGE and LC-MS analysis.

### 4.8. LC-MS Analysis

The sericin peptide library or the sample from CoIP was filtered using an ultrafiltration tube with molecular retention of 10 kDa (GE, Boston, MA, USA). The filtrate was collected, freeze-dried, re-dissolved in 0.1% (*w*/*v*) trifluoroacetic acid, and desalted using a desalination column (GE, Boston, MA, USA). Samples were then freeze-dried and redissolved in 0.1% (*v*/*v*) formic acid solution. Finally, the data were collected using a Thermo Scientific™ liquid chromatography–mass spectrometer (Thermo Fisher) and processed using Byonic v2.16.11 (Protein Metrics, San Carlos, CA, USA) software.

### 4.9. Molecular Docking, Molecular Dynamics, and Calculation of Molecular Free Energy

The spatial conformation, static charge, and energy of the TS263/1000 ligand were optimized to simulate the physiological environment using Schrödinger2021-3 software. The TGFβR2 receptor (ID:1KTZ) downloaded from the PDB database was modified using Maestro software (version 12.9) by removing water molecules, adding hydrogen atoms, and optimizing the force field. Subsequently, the centroid of the eutectic ligand in the TGFβR2 crystal structure was selected as the center of the active pocket for docking with the TS-263/1000 ligand using the SP precision algorithm.

In the molecular dynamics study, the ligand and receptor were described by the ff14SB protein force field, and the Na^+^/Cl^−^ system was used to balance the charge after adding the cross-section of the octahedral TIP3P solvent cassette at a distance of 10 Å in the docking system. The energy of the docking system with constant volume was then optimized using the steepest descent method of 2500 steps and conjugate gradient method, the steepest descent method of 2500 steps, and rapidly heated from 0 K to 298.15 K within 200 ps and was maintained at 298.15 K. To ensure uniform distribution of solvent molecules in a solvent box, the docking system was run using the NVT (isothermal and isobaric) ensemble simulation for 500 ps and the NPT (isothermal and isobaric) ensemble simulation for 500 ps. Finally, the composite system was subjected to the NPT (isothermal and isobaric) ensemble simulations under periodic boundary conditions. The parameters used in the process of dynamic calculation were as follows: truncation distance of non-bond force at 10 Å; long-range electrostatic interaction; particle mesh Ewald (PME) method [35]; limitation of the length of the hydrogen bond (SHAKE method) [36]; and temperature control (Langevin algorithm) [37]. 

To calculate the binding free energy between the ligand and receptor, the MM/GBSA method was used to calculate the MD trajectory for 40–50 ns [38]. The calculation formula is as follows:

$${\Delta G}_{bind}\;=\;{\Delta G}_{complex}\;-\;\left({\Delta G}_{receptor}\;+\;{\Delta G}_{ligand}\right)\;=\;{\Delta E}_{internal}\;+\;{\Delta E}_{VDW}\;+\;{\Delta E}_{elec}{\;+\;\Delta G}_{GB}\;+\;{\Delta G}_{SA}$$

In this formula, ΔE_internal_, ΔE_VDW_, ΔE_elec_, ΔE_GB_, and ΔE_SA_ represent the internal energy, van der Waals interaction, electrostatic interaction, polar solvation-free energy, and non-polar solvation-free energy, respectively. The entropy change was omitted in this study because of its low accuracy and excessive consumption of computing resources [39].

### 4.10. Statistical Analysis

Data are presented as the mean ± standard error of the mean (SEM), and Student’s *t*-test was used for statistical analysis in this study. Statistical significance was set at *p* < 0.05.

## 5. Conclusions

In this study, we established a novel method for the rapid screening of TTPs by combining transcriptomics with CoIP technology. This method can greatly enhance the screening efficiency of TTPs and enable us to screen TS263/1000 from the sericin peptide library. TS263 can promote HaCat and HFF-1 cells to synthesize extracellular matrix proteins in a time- and concentration-dependent manner. We believe that this method can provide a reference for screening other functional peptides, proteins, nucleic acids, lipids, and small-molecule drugs obtained from natural resources.

## Figures and Tables

**Table 1:** Calculation of relative content of extracellular matrix protein.

Name	Col1A1	Elastin
Null	1.11 ± 0.09	1.33 ± 0.13 ^1^
Ser-P	1.91 ± 0.11	2.30 ± 0.10

^1^ The calculation formula is as Equation (1).

**Table 2:** Calculation of relative content of extracellular matrix protein.

Name	Col1A1	Elastin
Null	1.19 ± 0.04	0.99 ± 0.04
TS263	2.08 ± 0.03	1.94 ± 0.05
TS1000	1.73 ± 0.05	1.59 ± 0.03

**Table 3:** Prediction of binding free energy and its components using MM/GBSA method.

Scheme	TS263_TGFβR2	TS1000_TGFβR2
ΔE_vdw_	−27.85 ± 3.91	−24.69 ± 3.69
ΔE_elec_	−234.13 ± 6.41	−104.13 ± 7.04
ΔG_GB_	245.64 ± 7.42	117.90 ± 6.08
ΔG_SA_	−5.92 ± 0.27	−4.60 ± 0.06
ΔG_bind_	−22.26 ± 2.22	−15.52 ± 2.55

ΔE_vdw_, van der Waals energy; ΔE_elec_, electrostatic energy; ΔG_GB_, electrostatic contribution to solvation; ΔG_SA_, nonpolar contribution to solvation; ΔG_bind_, binding free energy. The unit for all values in the table is kcal/mol.

**Table 4:** RT-PCR analysis of genes related to the synthesis or degradation of extracellular matrix proteins.

Name	Null	TS263
Col1A1	4.70 ± 0.08 × 10^−1^	5.68 ± 0.01 × 10^−1^
Col3A1	1.51 ± 0.06 × 10^−2^	2.27 ± 0.11 × 10^−2^
Elastin	0.71 ± 0.01 × 10^0^	2.41 ± 0.11 × 10^0^
HYAL1	2.43 ± 0.17 × 10^−2^	5.92 ± 0.80 × 10^−2^
HAS3	6.40 ± 0.12 × 10^−1^	8.22 ± 0.11 × 10^−1^
TIMP3	4.81 ± 0.10 × 10	6.19 ± 0.13 × 10
MMP2	3.49 ± 0.01 × 10^−1^	2.28 ± 0.03 × 10^−1^
MMP9	2.40 ± 0.14 × 10^0^	1.16 ± 0.03 × 10^0^

**Table 5:** RT-PCR analysis of the key gene of JNK, ERK, P38, and TGF-β signaling pathway.

Name	Null	TS263
JNK	6.835 ± 1.40 × 10^−3^	7.365 ± 0.69 × 10^−3^
ERK	1.694 ± 0.15 × 10^−3^	1.776 ± 0.15 × 10^−3^
P38	5.596 ± 0.31 × 10^−1^	5.633 ± 0.55 × 10^−1^
Smad2	1.632 ± 0.02 × 10^−1^	1.955 ± 0.07 × 10^−1^

**Table 6:** Primers used in this study.

Name.	The Forward Primer	The Reverse Primer
Col1A1	5′-GCAGCCCTGGTGAAAATGGA-3′	5′-CAGCACCAGTAGCACCATCA-3′
Col3A1	5′-TGGAGGATGGTTGCACGAAA-3′	5′-ACAGCCTTGCGTGTTCGATA-3′
Elastin	5′-GGCCATTCCTGGTGGAGTTCC-3′	5′-AACTGGCTTAAGAGGTTTGCCT-3′
HYAL1	5′-CTTCAGCCCCAAGGTTGTCC-3′	5′-AAGCCTTGGGCCATATCGAG-3′
HAS3	5′-TCGGGGAAGAGTGCTCTCAG-3′	5′-GTGGATGAACTGGTAGCCCG-3′
TIMP3	5′-GTGGCACTCATTGCTTGTGG-3′	5′-CAAGGTGACGGGACTGGAAG-3′
MMP2	5′-ATCCAGACTTCCTCAGGCGG-3′	5′-TCCTGGCAATCCCTTTGTATGT-3′
MMP9	5′-GTACTCGACCTGTACCAGCG-3′	5′-AGAAGCCCCACTTCTTGTCG-3′
JNK	5′-TTGGAACACCATGTCCTGAA-3′	5′-ATGTACGGGTGTTGGAGAGC-3′
ERK	5′-CGTGTTTGAGTCAAGCCAGA-3′	5′-GCTGCAATGGTATCCACCTT-3′
P38	5′-GCCCCAGTAGTCAGAAGCAG-3′	5′-TAGGGGCTGAAGAGAGGTGA-3′
Smad2	5′-CGAAATGCCACGGTAGAAAT-3′	5′-CCAGAAGAGCAGCAAATTCC-3′
GAPDH	5′-CCATGGGGAAGGTGAAGGTC-3′	5′-TGAAGGGGTCATTGATGGCA-3′

## Data Availability

Since the data needs to be used for other studies, we do not provide the original transcriptome sequencing data.

## Supplementary Materials

The following supporting information can be downloaded at: https://www.mdpi.com/article/10.3390/ijms24119425/s1.
