# Supplementary material for: A Novel Approach for Screening Sericin-Derived Therapeutic Peptides Using Transcriptomics and Immunoprecipitation
Source: Int J Mol Sci. 2023 May 29;24(11):9425. doi: 10.3390/ijms24119425 (PMC10253923; doi:10.3390/ijms24119425)
Supplement: Supplementary file 1 [file ijms-24-09425-s001.zip › ijms-2328755-supplementary.pdf]

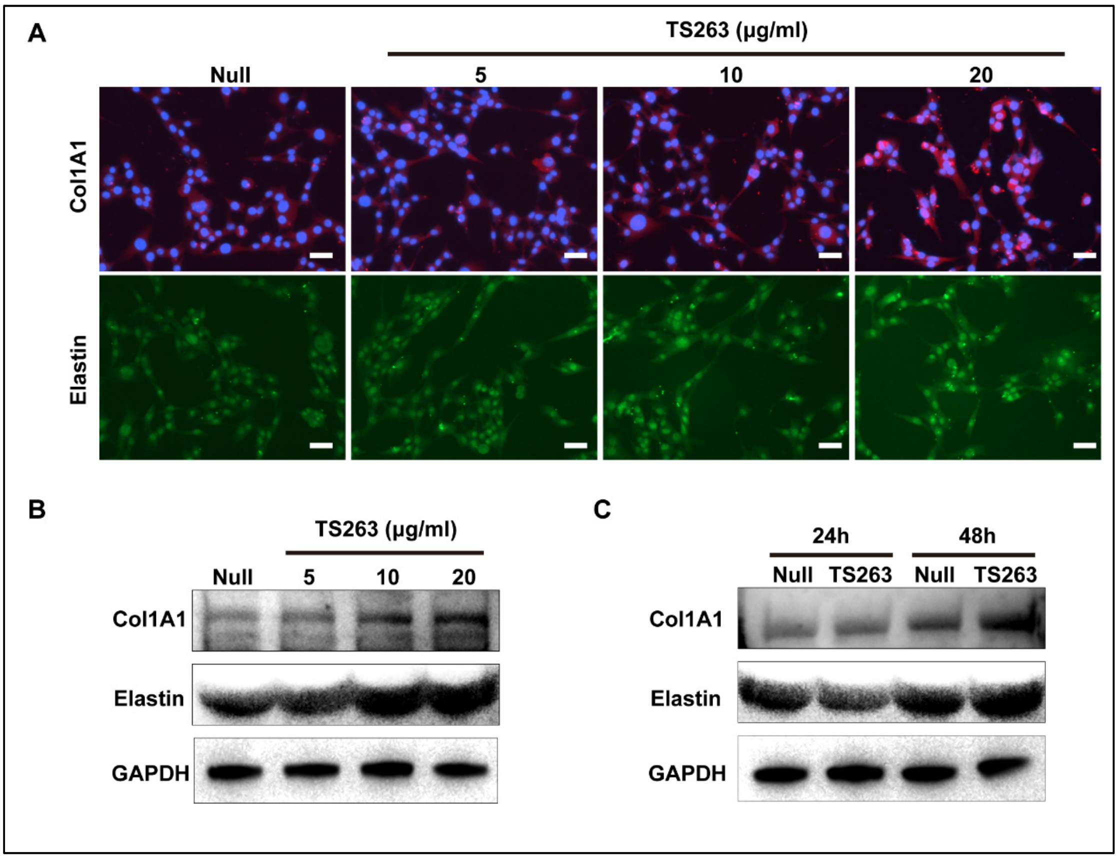

Figure S1. TS263 promotes the synthesis of extracellular matrix in human foreskin fibroblasts. (A) TS263 can promote HFF-1 cells to synthetize collagen I and elastin in a concentration dependent manner. (B), (C) Detection of collagen I and elastin in the medium at different concentrations or different times. All experiments were independently repeated three times.
